# Risk factors for overweight and overfatness in rural South African children and adolescents

**DOI:** 10.1093/pubmed/fdv016

**Published:** 2015-03-04

**Authors:** E. Craig, J.J. Reilly, R. Bland

**Affiliations:** 1 Section of Human Nutrition, University of Glasgow, Yorkhill Hospitals, Glasgow G3 8SJ, UK; 2 Africa Centre for Health and Population Studies, University of KwaZulu-Natal, Mtubatuba, KwaZulu-Natal 3935, South Africa; 3 School of Psychological Sciences and Health, University of Strathclyde, Glasgow G1 1QE, UK; 4 Royal Hospital for Sick Children, University of Glasgow, Glasgow G3 8SJ, UK

**Keywords:** children, obesity, social determinants

## Abstract

**Background:**

To determine risk factors for overweight/overfatness in children and adolescents from rural KwaZulu-Natal, South Africa.

**Methods:**

Anthropometric data were collected from a cross-sectional sample (*n* = 1519, ages 7, 11 and 15 years) and linked to demographic information (*n* = 1310 and *n* = 1317 in overweight and overfat analyses, respectively). Candidate risk factors for overweight/overfatness were identified and tested for associations with overweight (BMI-for-age >+1SD, WHO reference) and overfatness (>85th centile body fatness, McCarthy reference) as outcomes. Associations were examined using simple tests of proportions (*χ*^2^/Mann–Whitney *U* tests) and multivariable logistic regression.

**Results:**

Sex was a consistent variable across both analyses; girls at significantly increased risk of overweight and overfatness (overweight: *n* = 180, 73.9 and 26.1% females and males, respectively (*P* < 0.0001); overfat: *n* = 187, 72.7 and 27.3% females and males, respectively (*P* < 0.0001)). In regression analyses, sex and age (defined by school grade) were consistent variables, with boys at lower risk of overweight (adjusted odds ratio (AOR) 0.40 (confidence interval (CI) -0.28–0.57)) and risk of overweight increasing with age (AOR 0.65 (CI- 0.44–0.96), 0.50 (CI-0.33–0.75) and 1.00 for school grades 1, 5 and 9, respectively). Results were similar for overfatness.

**Conclusions:**

This study suggests that pre-adolescent/adolescent females may be the most appropriate targets of future interventions aimed at preventing obesity in rural South Africa.

## Introduction

Childhood obesity is one of the most serious public health challenges currently facing individuals, communities and governments throughout the world and has reached epidemic proportions.^1–3^ Often considered an issue primarily affecting high-income, developed countries, the childhood obesity epidemic is now a global problem, and its impact is increasingly evident in low- and middle-income countries (LMIC), particularly in urban settings.^4^

Risk factors for overweight and obesity consistently highlighted in reviews of this subject are often those directly related to individual energy balance such as diet, physical activity and sedentary behaviour.^5–7^ However, obesity is a complex and multifactorial condition which may be determined by a combination of individual-, household- or community-level factors.^4,8,9^ Despite this, research to date has concentrated on individual factors with very little evidence on ‘higher level’ influences on obesity.^10,11^

The majority of aetiological research on childhood obesity to date has been conducted in high-income countries; therefore, there is a substantial lack of data on the risk factors for child and adolescent obesity from middle-income countries like South Africa,^2,12^ and within LMIC there is a distinct lack of evidence from rural populations.^4^ In rural KwaZulu-Natal, despite extremely high prevalence of HIV,^13,14^ prevalence of adult overweight (including obesity) is now very high, particularly in women,^13,14^ but risk factors for obesity in children and adolescents have not been examined.

Interventions to prevent childhood obesity have had disappointing results, and this may be in part because the aetiology of obesity is poorly understood and prevention programmes have not targeted appropriate behaviours nor adequately engaged communities being studied.^15–19^ Further research on risk factors for obesity in rural and LMIC settings is needed to inform the development of effective interventions.^4,20^ Understanding risk factors for obesity is regarded as the key to prevention, both by identifying high-risk groups and high-risk behaviours;^9,16,21^ however, as noted above, there is a dearth of evidence on risk factors from sub-Saharan Africa and from rural populations.^22–25^

This cross-sectional study aimed to determine risk factors of overweight and obesity in a rural, African area; prevalence data are published elsewhere.^26^

## Materials and methods

### Study setting

This study was conducted within the demographic surveillance area (DSA) of the Africa Centre (www.africacentre.com) in rural KwaZulu-Natal, South Africa. The Africa Centre is situated in an area with a high HIV prevalence (29% in adults age 15–49 years).^13^ Despite the common belief that HIV infection leads to high levels of underweight, a previous study in this area, conducted before the widespread introduction of antiretroviral therapy, found that 58% of adults (woman aged 25–49 and men aged 25–54) were overweight, including 32% obese.^14^ The Africa Centre operates a large demographic and HIV surveillance of ∼92 000 individuals from 11 000 households, twice yearly, across a surveillance area of 438 km^2^. Data collected include family structure, household socioeconomic factors, household structure, education levels, births, deaths and migration within families. All homesteads, buildings and amenities including schools, water supplies and roads are mapped using a Geographic Information System (GIS). Each homestead within the Africa Centre DSA has been assigned a unique identifier, a 5 digit number, which identifies that particular homestead and is situated on a plaque on the outside wall of the homestead, this is called a Bounded Structure Identifier (BSID). During the consent process, participants were requested to note down their BSID and provide it with their returned consent form.

### Participants and anthropometric measures

Study participants were black Zulu children and adolescents recruited from local primary and secondary schools and enrolled in school grades 1, 5 and 9 (approximate ages 7, 11 and 15 years). All participating schools were within the Africa Centre DSA. All measurements (height, weight, MUAC and % body fat estimates) were carried out by trained field workers, and standard operating procedures were in adherence with WHO standards.^27,28^ Overweight (including obesity) was defined in the present study using the WHO 2007 BMI-for-age reference where overweight is classified as having a *z*-score of greater than +1SD.^29^ The descriptive statistics for BMI are classified as overweight and not overweight; overweight includes both overweight and obese, not overweight includes individuals who are either underweight or healthy weight.

Overfatness was defined using McCarthy 2006 body fat reference curves for children and adolescents based on bio-impedance (TANITA SC240MA). Overfatness in the present study was defined using cut-offs for excess fatness that were age and sex specific and defined as the 85th percentile of body fat % from the McCarthy reference.^30^ The descriptive statistics for body fat are classified as overfat and not overfat; overfat includes individuals who are overfat or obese and not overfat includes individuals who are either underfat or healthy (See Supplementary data for additional results obtained using Cole 2007 and Cole/IOTF 2000 reference).

### Statistical analyses

Individual, maternal and household factors were identified as exposures if previous studies in the literature had suggested that they may be associated with weight or fatness, or if it was believed that they may have the potential to affect weight or fat status in this particular population. This study was constrained to using variables available in the Africa Centre datasets (Africa Centre Demographic Information System (ACDIS)). Geographic variables (distance to nearest road and distance to nearest appropriate school) were available from the GIS. Variables were related to individual, maternal and household factors and therefore operated at different levels of the ecological model. All data were analysed using STATA 11.0.^31^

The descriptive analyses investigating tests of proportion included all potential risk factors (both individual and higher level). For the regression analyses, all potential risk factors were included at the univariable level and any which were significant, or which appeared to indicate a trend, were then entered simultaneously into a final multivariable model.

For each anthropometric measure, results of a descriptive analysis are included, followed by the univariable and multivariable regression.

### Ethics

Ethical approval was granted by the Biomedical Research Ethics Committee, University of KwaZulu-Natal (BE028/010). Written informed consent was obtained from parents or guardians including permission to access the information in the ACDIS and assent from the participant themselves.

## Results

A total of 1519 children participated in this cross-sectional study and had measurements taken.^26^ The present analyses are based on participants successfully matched to their unique ACDIS information using the unique BSID assigned to their homestead by the Africa Centre (*n* = 1310 for overweight analyses, 587 and 723 males and females, respectively, and *n* = 1317 for McCarthy overfat analyses, 590 and 727 males and females, respectively).

Table 1 presents the result of the descriptive analyses. Female sex was the only variable showing a significant association with being overweight using the BMI-based definition of overweight. One hundred and eighty subjects were overweight of whom 73.9 and 26.1% were females and males, respectively (*P* < 0.0001).

**Table 1:** Descriptive characteristics of overweight/not overweight and overfat/not overfat study participants, as defined by WHO 2007 BMI-for-age reference and McCarthy 2006 Body Fat reference^29,30^

*Characteristics*	*Overweight defined from BMI with WHO reference (*n* = 1310)*			*Overfat defined from bioelectrical impedance with the McCarthy reference (*n* = 1317)*		
	*Not overweight, *n* = 1130*	*Overweight, *n* = 180*	**χ*^2^ or Mann–Whitney *P*-value*	*Not overfat, *n* = 1130*	*Overfat, *n* = 187*	**χ*^2^ or Mann–Whitney *P*-value*
Sex						
Female	590 (52.21%)	133 (73.89%)	*χ*^2^ = 29.5001 *P* < 0.0001	591 (52.3%)	136 (72.73%)	*χ*^2^ = 27.0709 *P* < 0.0001
Male	540 (47.79%)	47 (26.11%)		539 (47.7%)	51 (27.27%)	
Mother's age at childbirth						
10–19	202 (23.76%)	26 (18.44%)	*χ*^2^ = 5.6415 *P* = 0.130	204 (23.8%)	24 (17.65%)	*χ*^2^ = 3.5127 *P* = 0.319
20–29	380 (44.71%)	68 (48.23%)		385 (44.92%)	64 (47.06%)	
30–39	229 (26.94%)	35 (24.82%)		226 (26.37%)	38 (27.94%)	
40+	39 (4.59%)	12 (8.51%)		42 (4.9%)	10 (7.35%)	
Missing	280	39		273	51	
Mother alive/dead						
Alive	573 (84.76%)	93 (86.11%)	*χ*^2^ = 0.1323 *P* = 0.716	575 (85.06%)	92 (84.40%)	*χ*^2^ = 0.0316 *P* = 0.859
Dead	103 (15.24%)	15 (13.89%)		101 (14.94%)	17 (15.6%)	
Missing	454	72		454	78	
Mother co-resident with child						
Yes	409 (60.5%)	61 (56.48%)	*χ*^2^ = 0.6272 *P* = 0.428	409 (60.5%)	62 (56.88%)	*χ*^2^ = 0.5131 *P* = 0.474
No	267 (39.5%)	47 (43.52%)		267 (39.5%)	47 (43.12%)	
Missing	454	72		454	78	
Mother's marital status						
Married	196 (27.11%)	40 (33.33%)	*χ*^2^ = 2.0235 *P* = 0.364	203 (27.92%)	34 (29.06%)	*χ*^2^ = 1.1717 *P* = 0.557
Single	472 (65.28%)	71 (59.17%)		466 (64.10%)	77 (65.81%)	
Widowed/separated	55 (7.61%)	9 (7.5%)		58 (7.98%)	6 (5.13%)	
Missing	407	60		403	70	
Number of individuals in household^^a^^						
1–5	219 (19.38%)	43 (23.89%)	*χ*^2^ = 2.3904 *P* = 0.303	230 (20.35%)	36 (19.25%)	*χ*^2^ = 0.1937 *P* = 0.908
6–15	518 (45.84%)	82 (45.56%)		514 (45.49%)	88 (47.06%)	
16+	393 (34.78%)	55 (30.56%)		386 (34.16%)	63 (33.69%)	
Missing	0	0		0	0	
Number of individuals under 18 in household						
1–5	521 (46.11%)	99 (55%)	*χ*^2^ = 6.7053 *P* = 0.082	527 (46.64%)	95 (50.8%)	*χ*^2^ = 1.2981 *P* = 0.730
6–10	250 (22.12%)	32 (17.78%)		245 (21.68%)	39 (20.86%)	
11–15	53 (4.69%)	11 (6.11%)		55 (4.87%)	9 (4.81%)	
16+	306 (27.08%)	38 (21.11%)		303 (26.81%)	44 (23.53%)	
Missing	169	28		0	0	
Median distance to nearest appropriate school (km)^^b^^	1.31 (0.78–1.97) (*n* = 1120)	1.33 (0.82–2.13) (*n* = 179)	Mann–Whitney = −0.507 *P* = 0.6120	1.34 (0.80–1.99)	1.27 (0.72–2.05)	Mann–Whitney = 0.823 *P* = 0.4104
Median distance to Level 1 road (km)^^c^^	7.49 (1.2–13.49) (*n* = 1120)	6.78 (1.2–14.2) (*n* = 179)	Mann–Whitney = 0.339 *P* = 0.7348	7.38 (1.22–13.42)	7.52 (1.15–14.42)	Mann–Whitney = 0.395 *P* = 0.6931
Water						
Piped	558 (70.81%)	87 (72.5%)	*χ*^2^ = 0.1442 *P* = 0.704	561 (70.92%)	85 (70.83%)	*χ*^2^ = 0.0004 *P* = 0.984
Other	230 (29.19%)	33 (27.50%)		230 (29.08%)	35 (29.17%)	
Missing	342	60		339	67	
Toilet						
Flush	33 (5.23%)	5 (5.26%)	*χ*^2^ = 0.4375 *P* = 0.804	33 (5.22%)	5 (5.21%)	*χ*^2^ = 0.1483 *P* = 0.929
Ventilation pit	96 (15.21%)	12 (12.63%)		95 (15.03%)	13 (13.54%)	
Other	502 (79.56%)	78 (82.11%)		504 (79.75%)	78 (81.25%)	
Missing	499	85		498	91	
Electricity						
Yes	593 (75.45%)	95 (78.51%)	*χ*^2^ = 0.5386 *P* = 0.463	595 (75.41%)	95 (78.51%)	*χ*^2^ = 0.5502 *P* = 0.458
No	193 (24.55%)	26 (21.49%)		194 (24.59%)	26 (21.49%)	
Missing	344	59		341	66	
Cooking fuel						
Electricity	451 (57.16%)	71 (58.68%)	*χ*^2^ = 1.2875 *P* = 0.525	451 (57.02%)	72 (59.5%)	*χ*^2^ = 2.4350 *P* = 0.296
Gas	46 (5.83%)	4 (3.31%)		47 (5.94%)	3 (2.48%)	
Wood/Coal/Other	292 (37.01%)	46 (38.02%)		293 (37.04%)	46 (38.02%)	
Missing	341	59		339	66	
Financial status^^d^^						
Poor	159 (20.23%)	26 (21.49%)	*χ*^2^ = 3.2317 *P* = 0.199	158 (20.03%)	84 (69.42%)	*χ*^2^ = 2.3585 *P* = 0.308
Just getting by	591 (75.19%)	85 (70.25%)		593 (75.16%)	28 (23.14%)	
Comfortable	36 (4.58%)	10 (8.26%)		38 (4.82%)	9 (7.44%)	
Missing	344	59		341	66	
Adult missed meal^^e^^						
Yes	28 (3.57%)	3 (2.48%)	*χ*^2^ = 0.3779 *P* = 0.539	28 (3.56%)	3 (2.48%)	*χ*^2^ = 0.3699 *P* = 0.543
No	756 (96.43%)	118 (97.52%)		759 (96.44%)	118 (97.52%)	
Missing	346	59		343	66	
Asset index^^f^^						
1 Poorest	157 (20.03%)	20 (16.81%)	*χ*^2^ = 5.2555 *P* = 0.262	158 (20.13%)	19 (15.83%)	*χ*^2^ = 2.0305 *P* = 0.730
2 ↓	170 (21.68%)	28 (23.53%)		171 (21.78%)	28 (23.33%)	
3 ↓	178 (22.7%)	19 (15.97%)		173 (22.04%)	24 (20%)	
4 ↓	175 (22.32%)	30 (25.21%)		173 (22.04%)	31 (25.83%)	
5 Wealthiest	104 (13.27%)	22 (18.49%)		110 (14.01%)	18 (15%)	
Missing	346	61		345	67	
Mother's highest school level						
Matriculation^g^ and above	152 (29.63%)	25 (29.76%)	*χ*^2^ = 0.2193 *P* = 0.974	147 (28.49%)	31 (37.8%)	*χ*^2^ = 4.9235 *P* = 0.177
Some secondary	180 (35.09%)	31 (36.9%)		183 (35.47%)	28 (34.15%)	
Some primary	118 (23%)	19 (22.62%)		119 (23.06%)	18 (21.95%)	
Never went to school	63 (12.28%)	9 (10.71%)		67 (12.98%)	5 (6.1%)	
Missing	617	96		614	105	
Mother's employment						
Employed	192 (33.33%)	32 (34.78%)	*χ*^2^ = 0.0748 *P* = 0.785	194 (33.39%)	32 (36.36%)	*χ*^2^ = 0.3020 *P* = 0.583
Not employed	384 (66.67%)	60 (65.22%)		387 (66.61%)	56 (63.64%)	
Missing	554	88		549	99	

^a^Household—Social group, with individuals as members. A narrow definition would restrict the term to groups of individuals who live and eat together, but ACDIS uses a wider definition also allowing for non-resident household members. We replace ‘share food, prepare and eat together’ by ‘largely share the same resources’ or ‘care for each other/would care for each other, if need be’.

^b^Nearest appropriate school—This refers to the nearest school appropriate for the child's current schooling level, i.e. for those in Grades 1 and 5, this is the nearest primary school and for those in Grade 9 it is the nearest secondary school.

^c^Level 1 road—This refers to a main national road. Level 2 roads are district roads and Level 3 roads are local roads.

^d^Financial status—This is based on the household's own perception of their financial status and therefore is a subjective measure. ‘Extremely poor’, ‘poor’, ‘just getting by’, ‘comfortable’ and ‘very comfortable’, recoded here to poor (including extremely poor and poor), just getting by and comfortable (including comfortable and very comfortable).

^e^Adult missed meal—This refers to whether an adult has needed to reduce the size of meals or completely miss a meal for financial reasons at any time within the past 12 months.

^f^Asset index—This is an objective measure using a wealth index developed for use with Demographic and Health Survey data (39). This index relates to assets owned by the household as well as amenities such as water, electricity and toilet facilities available. The scale is based on quintiles from 1 to 5 with 1 being the poorest and 5 being the wealthiest.

^g^Matriculation—This is often referred to as the school exit examination although it also relates to the minimum entry requirements for enrolment at university.

Similarly to the BMI-based definition of overweight, the only significant variable related to body fatness was sex with a significantly higher percentage of females classed as overfat than males: 187 subjects were overfat of whom 72.7 and 27.3% were females and males, respectively (*P* < 0.0001). The remaining ACDIS variables showed no significant association with overfatness.

In the univariable logistic regression investigating odds of being classed as overweight (see Table 2), female sex, higher school grade, maternal age at childbirth being >40 years and fewer than five individuals under 18 years of age in household were significantly associated with overweight with BMI as the outcome. A trend was also noted for the asset index with overweight increasing with wealth.

**Table 2:** Univariable and multivariable regression analysis of ACDIS variables to analyse risk of overweight, defined from BMI with the WHO 2007 BMI-for-age reference

*Overweight regression analysis*					
*Characteristics, *n* = 1310*	*Events (total)*	*Unadjusted (OR) (CI)*	**P**	*Adjusted (OR) (CI)*	**P**
Sex					
Female	133/723	1.00	—	1.00	—
Male	47/587	0.39 (0.27–0.55)	<0.0001	0.40 (0.28–0.57)	<0.0001
School grade					
9	81/409	1.00	—	1.00	—
5	46/450	0.46 (0.31–0.68)	<0.0001	0.50 (0.33–0.75)	0.001
1	53/451	0.54 (0.37–0.79)	0.001	0.65 (0.44–0.96)	0.032
Mother's age at childbirth					
10–19	26/228	1.00	—	1.00	—
20–29	68/448	1.39 (0.86–2.25)	0.181	1.46 (0.89–2.4)	0.135
30–39	35/264	1.19 (0.69–2.04)	0.534	1.05 (0.60–1.83)	0.871
40+	12/51	2.39 (1.11–5.14)	0.026	2.0 (0.90–4.45)	0.090
Missing	39/319	1.08 (0.64–1.84)	0.770	1.04 (0.58–1.84)	0.906
Number of individuals under 18 in household					
1–5	99/620	1.00	—	1.00	—
6–10	32/282	0.67 (0.44–1.03)	0.069	0.64 (0.41–0.99)	0.044
11–15	11/64	1.09 (0.55–2.16)	0.800	1.04 (0.51–2.1)	0.920
16+	38/344	0.65 (0.44–0.97)	0.037	0.59 (0.36–0.95)	0.031
Missing	—	—	—	—	—
Asset index^a^					
1 Poorest	20/177	1.00	—	1.00	—
2 ↓	28/198	1.29 (0.7–2.39)	0.412	1.19 (0.64–2.24)	0.584
3 ↓	19/197	0.84 (0.43–1.63)	0.601	0.78 (0.40–1.54)	0.478
4 ↓	30/205	1.35 (0.73–2.47)	0.336	1.26 (0.68–2.35)	0.465
5 Wealthiest	22/126	1.66 (0.86–3.19)	0.129	1.59 (0.81–3.13)	0.178
Missing	61/407	1.38 (0.81–2.37)	0.237	1.69 (0.94–3.04)	0.080

Mother alive/dead, Mother co-resident with child, Mothers marital status, Number of individuals in household, Median distance to nearest appropriate school, Median distance to Level 1 road, Water, Toilet, Electricity, Cooking fuel, Financial status, Adult missed meal, Mother's education, Mother's employment were all included in the univariable analysis but did not reach significance or indicate a trend so are not shown here.

^a^Asset index—This is an objective measure using a wealth index developed for use with Demographic and Health Survey data (39). This index relates to assets owned by the household as well as amenities such as water, electricity and toilet facilities available. The scale is based on quintiles from 1 to 5 with 1 being the poorest and 5 being the wealthiest.

In the multivariable analysis, maternal age and the trend apparent for the asset index failed to reach significance. However, compared with males, females were twice as likely to be overweight (adjusted odds ratio (AOR) 1.00 and 0.4 for females and males, respectively), as were individuals in Grade 9 compared with those in Grades 5 and 1 (AOR 0.65, 0.50 and 1.00 Grade 1, 5 and 9 respectively). Having fewer individuals aged <18 in households was also associated with being overweight (AOR 1.00, 0.64, 1.04, and 0.59 for 1–5, 6–10, 11–15 and 16+ individuals, respectively).

Similarly, univariable logistic regression investigating odds of being classed as overfat (Table 3) found female sex and higher school grade to be significant with a trend observed with increasing maternal age at childbirth. Higher maternal education was also associated with being overfat.

**Table 3:** Univariable and multivariable regression analysis of ACDIS variables to analyse risk of overfatness, defined from bioelectrical impedance measures with the McCarthy *et al.* 2006 body fat-for-age reference^30^

*Overfat regression analysis*					
*Characteristics, *n* = 1317*	*Events (total)*	*Unadjusted (OR) (CI)*	**P**	*Adjusted (OR) (CI)*	**P**
Sex					
Female	136/727	1.00	—	1.00	—
Male	51/590	0.41 (0.29–0.58)	<0.0001	0.40 (0.28–0.57)	<0.0001
School grade					
9	72/418	1.00	—	1.00	—
5	32/451	0.37 (0.24–0.57)	<0.0001	0.38 (0.25–0.60)	<0.0001
1	83/448	1.09 (0.77–1.55)	0.618	1.2 (0.83–1.72)	0.329
Mother's age at childbirth					
10–19	24/228	1.00	—	1.00	—
20–29	64/449	1.41 (0.86–2.33)	0.174	1.62 (0.97–2.73)	0.067
30–39	38/264	1.43 (0.83–2.46)	0.199	1.51 (0.85–2.68)	0.155
40+	10/52	2.02 (0.9–4.54)	0.088	2.73 (1.13–6.59)	0.026
Missing	51/324	1.59 (0.95–2.67)	0.080	1.65 (0.92–2.95)	0.095
Mother's education					
Matriculation^a^ and above	31/178	1.00	—	1.00	—
Some secondary	28/211	0.73 (0.42–1.26)	0.257	0.74 (0.42–1.31)	0.297
Some primary	18/137	0.72 (0.38–1.35)	0.301	0.64 (0.33–1.24)	0.189
No schooling	5/72	0.35 (0.13–0.95)	0.039	0.30 (0.11–0.86)	0.025
Missing	105/719	0.81 (0.52–1.26)	0.350	0.78 (0.47–1.3)	0.338

Mother alive/dead, Mother co-resident with child, Mothers marital status, Number of individuals in household, Number of individuals under 18 in household, Median distance to nearest appropriate school, Median distance to Level 1 road, Water, Toilet, Electricity, Cooking fuel, Financial status, Adult missed meal, Asset index, Mother's employment were all included in the univariable analysis but did not reach significance or indicate a trend so are not shown here.

^a^Matriculation—This is often referred to as the school exit examination although it also relates to the minimum entry requirements for enrolment at university.

In multivariable analysis, sex, school grade and mother's education all remained significant with the trend apparent for maternal age at childbirth reaching significance. Compared with males, females were over twice as likely to be overfat (AOR 1.00 and 0.40 for females and males, respectively), as were those in Grade 9 compared with those in Grade 5 (AOR 1.00 versus 0.50, respectively). Compared with children of mothers who were under 19 years of age at childbirth, those whose mothers who were over 40 years were almost 3 times more likely to be overfat (AOR 1.00 and 2.73 for under 19 and over 40, respectively), and those whose mothers had completed matriculation (the school exit examination) were 3 times more likely to be overfat compared with those whose mothers had not been to school at all (AOR 1.00 and 0.30 for Matriculation and No schooling, respectively) (See Supplementary data for additional results obtained using Cole 2007 and Cole/IOTF 2000 reference).

## Discussion

### Main findings of this study

Few studies have carried out analyses of risk factors for child and adolescent obesity from sub-Saharan Africa. Regardless of how the analyses were undertaken, the consistent risk factors for obesity which emerged from the present study were female sex and higher school grade. Few of the higher level candidate risk factors were associated with overweight or overfat, and some candidate risk factors that might have been expected to be associated with overweight or overfat from other studies in the developed and developing world (for example, measures of socioeconomic status) appeared to be relatively unimportant in this population. Our results may suggest that prevention strategies to prevent overweight and obesity in this population should target girls, at a point before overweight/overfat become common. There appears to be a strong cultural acceptance of overweight/obesity in this population, and future community-wide education may be warranted to tackle the belief that overweight indicates health and wealth and is therefore desirable, especially in females. A further mechanism whereby more females may be at risk of overweight is that it is more acceptable and encouraged for boys to participate in sport, whereas girls may be expected to stay home and carry out household chores or look after children—an increase in sports availability to girls may be beneficial.

### What is already known on this topic

To the best of the authors' knowledge, only a handful of studies have been carried out in South Africa with the aim of investigating socioeconomic predictors of anthropometric status.^9,32–35^ These studies all found socioeconomic status to be significantly associated with weight status. It is unclear exactly why these associations were not shown in our sample; however, it may be due to the different (rural) population and setting of the present study compared with previous studies and possibly a result of a lack of variation in candidate risk factors and homogeneity in the present sample.

A recent multilevel analysis of 2100 children who were part of the early childhood longitudinal study in the USA assessed the ecological influences on obesity in early childhood.^36^ The study found that child and family factors accounted for 71% of the variance in overweight and obesity, while school- and community-level factors accounted for 27 and 2%, respectively. The authors suggest that these results imply interventions should firstly focus on factors relating to the child and family, then at the school level and finally at the community level. It is not clear whether the results would be the same in a LMIC, although the results of the present study also show individual level factors to have the most consistent effect. Other studies have also noted a link between maternal, child and family factors and risk of overweight and obesity.^11,37^

### What this study adds

There have been few studies of child and adolescent obesity risk factors in rural populations from the developing world, and the concept of analysing both individual- and higher level influences is novel in this under-researched but important population. This study also benefits from a relatively large sample size of children and adolescents. A further strength was use of a body composition measure as an outcome, in addition to BMI, and it was hypothesized that this may have revealed associations not detected by use of BMI alone.^38^

Alternative outcome measures can be used to define overweight and obesity, and these were available in the present study. Further analyses (not shown here) were also carried out using the Cole 2007 and IOTF 2000 BMI-based definitions of overweight and obesity and similar results were found, where sex and age were the only significant factors in the multivariable regression. This may be taken to suggest that present study results would be similar regardless of the outcome measure used.

### Limitations of this study

Household-level data were not available for every participant as some did not provide a valid household identifier (BSID) at the time of anthropometric measurement, and therefore, this made it difficult to match them to their data (although every effort was taken to match each individual via other methods including using their mother's and father's name). Where an individual had not been matched, analysis of higher level candidate risk factors could not be carried out, and therefore, the present analysis does not include all 1519 initially enrolled in the study on whom anthropometric measurements were taken.^26^

There is also the possibility that we may not have had the ‘right’ candidate risk factors in the present study or that the measures of the risk factors available were not measured with sufficient precision to be associated with overweight or overfat. A relatively recent review of systematic reviews of risk factors for childhood obesity by Monasta *et al.*^22^ found maternal smoking, breastfeeding, infant size and growth, short sleep duration and television viewing to be the risk factors supported by better-quality reviews. This review emphasized the difficulty of establishing causal associations and suggested early-life interventions to help improve the knowledge of both protective and risk factors. These early-life risk factors were not available in the present study, either because they had not been measured or because there was insufficient data to justify their inclusion; however, it may be the case that some of these risk factors (early-life TV exposure or short sleep duration) would not be applicable in this rural population. Further research would be required to explore the effect of these factors in the present population. Pubertal status was not assessed in the present study, but given that it has been highlighted as a possible risk factor in previous work,^9^ it may be beneficial to include this measure in any future research to further understand the change in weight and fatness that occurs in girls between 7 and 15 years. Further, given that the present population is in a state of transition, the present results may not apply in several years time when the area may be at a new stage of economic development.

### Conclusions

At present, the most justifiable target of obesity prevention efforts in rural South African children and adolescents would appear to be girls, and this may be urgent given the relatively high prevalence of overweight/overfatness present in the older girls and also in adult women in the area.^14^ Further research is required to explain the mechanism by which girls are at higher risk of overweight and overfat than boys in this population.

## Supplementary data

Supplementary data are available at the *PUBMED* online.

Supplementary Data
