# Supplementary material for: Risk factors for overweight and overfatness in rural South African children and adolescents
Source: J Public Health (Oxf). 2015 Mar 4;38(1):24–33. doi: 10.1093/pubmed/fdv016 (PMC4750520; doi:10.1093/pubmed/fdv016)
Supplement: Supplementary Data [file supp_38_1_24__index.html]

Risk factors for overweight and overfatness in rural South African children and adolescents — Supplementary Data 

# Risk factors for overweight and overfatness in rural South African children and adolescents

## Supplementary Data

Supplementary Data

**Files in this Data Supplement:**

- Supplementary appendix1 - docx file
- Supplementary appendix2 - docx file
