# Supplementary material for: Risk factors for overweight and overfatness in rural South African children and adolescents
Source: J Public Health (Oxf). 2015 Mar 4;38(1):24–33. doi: 10.1093/pubmed/fdv016 (PMC4750520; doi:10.1093/pubmed/fdv016)
Supplement: Supplementary Data [file supp_fdv016_fdv016supp_appendix1.docx]

Appendix 1. Descriptive characteristics of overweight/obese and not overweight/obese study participants, as defined by IOTF 2000 and Cole 2007, BMI-for-age reference ([Cole et al., 2000](#_ENREF_76), [Cole et al., 2007](#_ENREF_77))

| ***IOTF*** | | | |
| --- | --- | --- | --- |
| **Characteristic**  **(N=1292)** | **Not Overweight**  **n=1163** | **Overweight**  **n=129** | **X^2^ or Mann Whitney**  **P value** |
| **Sex** |  |  |  |
| Female | 612 (52.62%) | 104 (80.62%) | X^2^ = 36.8416 p<0.0001 |
| Male | 551 (47.38%) | 25 (19.38%) |  |
| **Mother’s Age at Childbirth** |  |  |  |
| 10-19 | 204 (23.18%) | 24 (24%) | X^2^ = 5.5778 p = 0.134 |
| 20-29 | 394 (44.77%) | 47 (47%) |  |
| 30-39 | 242 (27.50%) | 20 (20%) |  |
| 40+ | 40 (4.55%) | 9 (9%) |  |
| Missing | 283 | 29 |  |
| **Mother Alive/Dead** |  |  |  |
| Alive | 591 (84.91%) | 68 (87.18%) | X^2^ = 0.2846 p = 0.594 |
| Dead | 105 (15.09%) | 10 (12.82%) |  |
| Missing | 467 | 51 |  |
| **Mother Co-Resident with child** |  |  |  |
| Yes | 416 (59.77%) | 48 (61.54%) | X^2^ = 0.0913 p = 0.762 |
| No | 280 (40.23%) | 30 (38.46%) |  |
| Missing | 467 | 51 |  |
| **Mother’s Marital Status** |  |  |  |
| Married | 204 (27.24%) | 29 (33.72%) | X^2^ = 1.7313 p = 0.421 |
| Single | 489 (65.29%) | 52 (60.47%) |  |
| Widowed/Separated | 56 (7.48%) | 5 (5.81%) |  |
| Missing | 414 | 43 |  |
| **Number of Individuals in Household^1^** |  |  |  |
| 1-5 | 225 (19.35%) | 32 (24.81%) | X^2^ = 2.2997 p = 0.317 |
| 6-15 | 533 (45.83%) | 57 (44.19%) |  |
| 16+ | 405 (34.82%) | 40 (31.01%) |  |
| Missing | 0 | 0 |  |
| **Number of individuals Under 18 in Household** |  |  |  |
| 1-5 | 543 (46.69%) | 68 (52.71%) | X^2^ = 4.2199 p = 0.239 |
| 6-10 | 252 (21.67%) | 26 (20.16%) |  |
| 11-15 | 55 (4.73%) | 9 (6.98%) |  |
| 16+ | 313 (26.91%) | 26 (20.16%) |  |
| Missing | 0 | 0 |  |
| **Median Distance to Nearest Appropriate School (km)^2^** | 1.31 (0.77-1.94)(n=1153) | 1.35 (0.83-2.27)(n=128) | Mann Whitney = -1.417 p = 0.1564 |
| **Median Distance to Level 1 Road (km)^3^** | 7.43 (1.20-13.5)n=1153 | 7.14 (1.29-14.07)n=128 | Mann Whitney = 0.099 p = 0.9208 |
| **Water** |  |  |  |
| Piped | 573 (70.74%) | 62 (73.81%) | X^2^ = 0.3483 p = 0.555 |
| Other | 237 (29.26%) | 22 (26.19%) |  |
| Missing | 353 | 45 |  |
| **Toilet** |  |  |  |
| Flush | 34 (5.25%) | 4 (5.88%) | X^2^ = 1.2249 p = 0.542 |
| Ventilation Pit | 99 (15.28%) | 7 (10.29%) |  |
| Other | 515 (79.48%) | 57 (83.82%) |  |
| Missing | 515 | 61 |  |
| **Electricity** |  |  |  |
| Yes | 612 (75.74%) | 67(78.82%) | X^2^ = 0.4007 p = 0.527 |
| No | 196 (24.26%) | 18(21.18%) |  |
| Missing | 355 | 44 |  |
| **Cooking Fuel** |  |  |  |
| Electricity | 468 (57.71%) | 49 (57.65%) | X^2^ = 3.3950 p = 0.183 |
| Gas | 46 (5.67%) | 1 (1.18%) |  |
| Wood/Coal/Other | 297 (36.62%) | 35 (41.18%) |  |
| Missing | 352 | 44 |  |
| **Financial Status^4^** |  |  |  |
| Poor | 164 (20.27%) | 20 (23.53%) | X^2^ = 1.4743 p = 0.478 |
| Just Getting By | 606 (74.91%) | 59 (69.41%) |  |
| Comfortable | 39 (4.82%) | 6 (7.06%) |  |
| Missing | 354 | 44 |  |
| **Adult Missed Meal^5^** |  |  |  |
| Yes | 26 (3.23%) | 3 (3.53%) | X^2^ = 0.0225 p = 0.881 |
| No | 780 (96.77%) | 82 (96.47%) |  |
| Missing | 357 | 44 |  |
| **Asset Index^6^** |  |  |  |
| 1 Poorest | 160 (19.88%) | 14 (16.67%) | X^2^ = 4.2348 p = 0.375 |
| 2 ↓ | 174 (21.61%) | 18 (21.43%) |  |
| 3 ↓ | 180 (22.36%) | 13 (15.48%) |  |
| 4 ↓ | 181 (22.48%) | 24 (28.57%) |  |
| 5 Wealthiest | 110(13.66%) | 15 (17.86%) |  |
| Missing | 358 | 45 |  |
| **Mother’s Highest School Level** |  |  |  |
| Matriculation^7^ and above | 155 (29.03%) | 22 (38.60%) | X^2^ = 2.3371 p = 0.505 |
| Some Secondary | 194 (36.33%) | 17 (29.82%) |  |
| Some Primary | 121 (22.66%) | 12 (21.05%) |  |
| Never went to school | 64 (11.99%) | 6 (10.53%) |  |
| Missing | 629 | 72 |  |
| **Mother’s Employment** |  |  |  |
| Employed | 201 (33.72%) | 22 (33.85%) | X^2^ = 0.0004 p = 0.984 |
| Not Employed | 395 (66.28%) | 43 (66.15%) |  |
| Missing | 567 | 64 |  |
|  |  |  |  |

**(For definition of footnotes please see Table 1 in main manuscript)**
