# Supplementary material for: Risk factors for overweight and overfatness in rural South African children and adolescents
Source: J Public Health (Oxf). 2015 Mar 4;38(1):24–33. doi: 10.1093/pubmed/fdv016 (PMC4750520; doi:10.1093/pubmed/fdv016)
Supplement: Supplementary Data [file supp_fdv016_fdv016supp_appendix2.docx]

Appendix 2

Univariable and multivariable regression analysis of ACDIS variables to analyse risk of overweight/obese, defined by Cole 2007/IOTF 2000 BMI-for-age reference ([Cole et al., 2007](#_ENREF_77), [Cole et al., 2000](#_ENREF_76))

| ***Cole/IOTF Regression Analysis*** | | | | | |
| --- | --- | --- | --- | --- | --- |
| **Characteristic**  **N=1292** | **Events (Total)** | **Unadjusted (OR) (CI)** | **p** | **Adjusted**  **(OR)(CI)** | **p** |
| **Sex** |  |  |  |  |  |
| Female | 104/716 | 1.00 | - | 1.00 | - |
| Male | 25/576 | 0.27  (0.17-0.42) | <0.0001 | 0.29  (0.19-0.46) | <0.0001 |
| **School Grade** |  |  |  |  |  |
| 9 | 67/391 | 1.00 | - | 1.00 | - |
| 5 | 34/450 | 0.40  (0.26-0.61) | <0.0001 | 0.42  (0.27-0.66) | <0.0001 |
| 1 | 28/451 | 0.32  (0.20-0.51) | <0.0001 | 0.37  (0.23-0.59) | <0.0001 |
| Missing | 43/457 | 0.73  (0.44-1.2) | 0.219 | ― | ― |
| **Number of Individuals in Household^1^** |  |  |  |  |  |
| 1-5 | 32/257 | 1.00 | - | 1.00 | - |
| 6-15 | 57/590 | 0.75  (0.47-1.19) | 0.225 | 0.75  (0.47-1.21) | 0.243 |
| 16+ | 40/445 | 0.69  (0.42-1.14) | 0.147 | 0.77  (0.46-1.27) | 0.303 |
| Missing | - | - | - | - | - |

**(For definition of footnotes please see Table 1 in main manuscript)**
